# Death receptor 5 is required for intestinal stem cell activity during intestinal epithelial renewal at homoeostasis

**DOI:** 10.1038/s41419-023-06409-4

**Published:** 2024-01-10

**Authors:** Jianbo Liu, Kaixuan Liu, Ying Wang, Ziru Shi, Runze Xu, Yundi Zhang, Jingxin Li, Chuanyong Liu, Bing Xue

**Affiliations:** Department of Physiology and Pathophysiology, School of basic medical science, Cheeloo College of Medicine, Shandong University, Jinan, China

**Keywords:** Stem-cell research, Physiology

## Abstract

Intestinal epithelial renewal, which depends on the proliferation and differentiation of intestinal stem cells (ISCs), is essential for epithelial homoeostasis. Understanding the mechanism controlling ISC activity is important. We found that death receptor 5 (DR5) gene deletion (DR5^-/-^) mice had impaired epithelial absorption and barrier function, resulting in delayed weight gain, which might be related to the general reduction of differentiated epithelial cells. In DR5^-/-^ mice, the expression of ISC marker genes, the number of Olfm4^+^ ISCs, and the number of Ki67^+^ and BrdU^+^ cells in crypt were reduced. Furthermore, DR5 deletion inhibited the expression of lineage differentiation genes driving ISC differentiation into enterocytes, goblet cells, enteroendocrine cells, and Paneth cells. Therefore, DR5 gene loss may inhibit the intestinal epithelial renewal by dampening ISC activity. The ability of crypts from DR5^-/-^ mice to form organoids decreased, and selective DR5 activation by Bioymifi promoted organoid growth and the expression of ISC and intestinal epithelial cell marker genes. Silencing of endogenous DR5 ligand TRAIL in organoids down-regulated the expression of ISC and intestinal epithelial cell marker genes. So, DR5 expressed in intestinal crypts was involved in the regulation of ISC activity. DR5 deletion in vivo or activation in organoids inhibited or enhanced the activity of Wnt, Notch, and BMP signalling through regulating the production of Paneth cell-derived ISC niche factors. DR5 gene deletion caused apoptosis and DNA damage in transit amplifying cells by inhibiting ERK1/2 activity in intestinal crypts. Inhibition of ERK1/2 with PD0325901 dampened the ISC activity and epithelial regeneration. In organoids, when Bioymifi’s effect in activating ERK1/2 activity was completely blocked by PD0325901, its role in stimulating ISC activity and promoting epithelial regeneration was also eliminated. In summary, DR5 in intestinal crypts is essential for ISC activity during epithelial renewal under homoeostasis.

## Introduction

The intestinal epithelium serves as a place for digestion, absorption, and barrier function [1]. Due to mechanical, chemical, and biological insults from the intestinal cavity, intestinal epithelial cells constantly detach from the apex of intestinal villi [2, 3]. Intestinal stem cells (ISCs) drive intestinal epithelial cell renewal through constant proliferation and differentiation to maintain epithelial homoeostasis [4]. The crypt base columnar (CBC) cells marked by leucine-rich-repeat-containing G protein-coupled receptor 5 (Lgr5) are the actively ISCs wedged between Paneth cells [5]. Lgr5^+^ ISCs divide to produce two daughters, one staying at the bottom of crypts to stabilize the ISCs pool, and the other giving rise to highly proliferative progenitor cells in the transit amplifying (TA) region of crypts, also known as TA cells, which proliferate and differentiate into all intestinal epithelium [6]. Another ISCs population, +4 cells are relatively quiescent and become more active following epithelial injury [7]. Niche cells, such as Paneth cells, trophocytes and subepithelial myofibroblasts produce niche factors to activate signalling such as Wnt, Notch, and bone morphogenetic protein (BMP) to orchestrate ISC activity [7–9]. ISC fate must be strictly controlled to ensure the epithelial homoeostasis under rapid turnover. However, the regulatory mechanism of ISC activity is complex and unclear.

Death receptor 5 (DR5), a member of the tumour necrosis factor receptor superfamily, can induce caspase-3-dependent apoptosis of tumoral cells after activation by the tumour necrosis factor-related apoptosis-inducing ligand (TRAIL) [10, 11]. In addition to proapoptotic effects in tumour cells [12, 13], DR5 activation has been reported to be involved in cell proliferation, survival, angiogenesis, etc., by regulating kinases activity [13, 14].

DR5 is expressed in colonic epithelial cells of humans and mice [15] and intestinal mucosal injury leads to change in DR5 expression in intestinal epithelial cells [16–18]. Deletion of DR5 gene increased susceptibility to dextran sulfate sodium**—**induced colitis in mice, while its activation increased the sensitivity of ISCs to chemotherapy-induced cell death [18, 19]. Unlike tumour cells, intestinal epithelial cells are not susceptible to TRAIL-induced apoptosis [20], suggesting potential non-apoptotic effects of DR5 at epithelial homoeostasis. However, the role of DR5 in intestinal epithelium is unclear. Our preliminary experiments identified the dominant expression of DR5 in ileal crypts, where ISCs, TA cells and Paneth cells are present. Given the central role of ISCs and Paneth cells in maintaining epithelial homoeostasis and the role of DR5 in cell proliferation and differentiation [11, 21], we wondered whether DR5 was involved in the regulation of intestinal epithelial homoeostasis. Therefore, we explored the role of DR5 in intestinal epithelial homoeostasis with DR5 gene deletion (DR5^-/-^) mice and its mechanism.

## Materials and methods

### Animals

Male adult (6–8 weeks, 20 g) C57BL/6 N mice were purchased from Beijing Vital River Laboratory Animal Technology Co., Ltd. DR5^-/-^ mice were purchased from Saiye Suzhou Biotechnology Co., Ltd. China, and the strain name was C57BL/6N-Tnfrsf10b^em1cyagen^. Animals were housed in a temperature-controlled room with a 12-h dark-light cycle. Mice had free access to food and water and were randomized grouped. The selection of sample size for animal experiments was carried out per as the preliminary experiments as well as similar research. All animal experiments were approved by the Ethics Committee of Laboratory Animal Medicine, School of Basic Medicine, Shandong University (Shandong, China).

### Assessment of body weight, food intake and faecal dry weight

We measured the body weight of the mice and their daily food intake every 3 days starting from 4 to 8 weeks of age. Body weight was expressed as a percentage change compared with the body weight on the first day at 4 weeks of age. Food intake was expressed as the average daily food mass per cage per mouse. Faeces of 8-week-old mice within 30 min were collected at 9 am to test the faecal dry weight, which was performed for 3 consecutive days and the mean value was taken.

### Hematoxylin and eosin (H&E) staining

The segments of ileum were removed to prepare the paraffin section (4 µm). Sections were oven-baked at 65 °C for 2 h, dewaxed with xylene and graded alcohol, and stained with hematoxylin and eosin. Villus length was measured by ImageJ software. Measurements were taken by two experienced observers blinded to the experimental protocol. At least 15 correctly aligned villi were measured for every section to obtain the mean value.

### Immunohistochemistry (IHC) and immunofluorescence (IF)

Paraffin slides were deparaffinized and rehydrated with xylene and graded alcohol. They were immersed in 0.01 M sodium citrate buffer (pH 6.0) in a microwave oven for antigen retrieval. Then, the sections were incubated with an endogenous peroxidase blocker for 30 min at room temperature to quench endogenous peroxidase activity. Triton X-100 (0.5%) was used to penetrate the cell membrane for IF staining. After the sections were rinsed with phosphate-buffered saline (PBS), they were blocked with goat serum (ZSGB-BIO, Beijing, China) for 1 h and then incubated with the appropriate antibody.

For IHC assays, the slide was incubated with the corresponding antibody (listed in Supplementary Table 1) at 4 °C overnight according to the experimental design. The next day immunodetection was performed by a two-step kit (ZSGB-BIO, Beijing, China) according to the manufacturer’s directions. Hematoxylin was used to counterstain the nuclei. The Ki67, BrdU, CDX2, lysozyme (Lyz), MUC2 and γH2AX- positive cells were counted by two experimenters blinded to the experimental design. At least 15 villi and/or 15 crypts were counted randomly for each slide and the mean value was calculated.

The primary antibodies and secondary antibodies for IF assays are shown in Supplementary Table 2. The slides were incubated with the primary antibody at 4 °C overnight and then incubated with the secondary antibody in a humid dark box at 37 °C for 60 min on the second day. 4’6-diamidino-2-phenylindole (DAPI) (Beyotime, Shanghai, China) was used to label the nuclei. The slides were observed under fluorescence microscope (Nikon, Japan).

### Alkaline phosphatase (ALP) staining and periodic acid–Schiff (PAS) staining

After baking and dewaxing, paraffin sections were dipped in double distilled water. Then, sections were stained with an ALP Stain Kit (Solarbio, Beijing, China) or a PAS Kit (Maixin, Fuzhou, China). Nuclear fast red was used to counterstain the nuclei in ALP staining, and haematoxylin was used to counterstain the nuclei in PAS staining. ALP staining was used to visualize the apical brush border of epithelial cells [22]. The goblet cells were labelled with PAS staining, and the number of PAS^+^ goblet cells in each section was counted by two experimenters blinded to the experimental design. At least 15 villi and crypts were counted randomly for each slide and the mean value was calculated.

### Terminal deoxynucleotidyl transferase-mediated dUTP nick-end labelling (TUNEL) assay and colocalization with Lyz

Detection of apoptosis in intestinal epithelial cells was performed using the one-step TUNEL Apoptosis Assay Kit (Keygen biotech, Jiangsu, China). After baking, dewaxing, and rehydrating, paraffin sections were dipped in PBS buffer. Then, the sections were incubated with proteinase K for 30 min and TdT enzyme reaction solution for 1 h at 37 °C. The sections were labelled with streptavidin-fluorescein for 30 min at 37 ℃. Rabbit monoclonal anti-Lyz (1:250; Abcam, Cambridge, UK) was used as the primary antibody to label Paneth cells. Nuclei were counterstained with DAPI. The slides were observed under a fluorescence microscope (Nikon, Japan). TUNEL^+^ Paneth cells and TUNEL^+^ TA cells were counted by two experimenters blinded to the experimental design. At least 20 crypts were counted randomly for each slide and the mean value was calculated.

### Transmission electron microscopy (TEM)

The ileal segment was removed and the lumen was flushed with ice-cold electron microscope fixing solution (2.5% glutaraldehyde in 0.1 M phosphate buffer) to clear the intestinal contents. Tissue was cut into 1 mm^3^ sections and fixed with 2.5% glutaraldehyde in 0.1 M phosphate buffer for 4 h followed by 1% osmium tetroxide in 0.1 M phosphate buffer for 1 h at 4 °C. Next, the tissue was dehydrated in graded acetone, and embedded with ethoxyline resin. Ultrathin sections (70 nm) were cut with an Ultra microtome (Leica, Cambridge, UK) and stained with 3% uranyl acetate and lead citrate. Finally, ultrastructural images were observed at the site of microvilli by a JEM-1200EX transmission electron microscope (Hitachi Electronic Company, Japan).

### Isolation of intestinal crypts

The ileal crypts were extracted rapidly following previous reports [23, 24]. Briefly, after mice were sacrificed, the ileal segment was cut ~1 cm away from the ileocecal region, and then an ileum segment ~10 cm was removed and quickly placed in ice-cold D-PBS buffer. The intestinal lumen was dissected longitudinally, and washed with cold D-PBS. Intestinal segments were cut into 3–4 mm pieces with scissors and transferred into 50 ml centrifuge tubes containing cold D-PBS. After vigorous washing was repeated ~20 times, the supernatant was removed, and this process was repeated 3–4 times. Thirty millilitres of ice-cold 2 mM EDTA (Invitrogen, California, USA) solution was added to resuspend the precipitate, which was shaken at 4 °C for 30 min, and then the supernatant was removed. After that, 20 ml of cold D-PBS was added, the supernatant was removed after repeated gentle pipetting ~15 times, and this process was repeated 1–2 times. The crypt suspension was passed through a 70 μm cell strainer 4–6 times. Crypts were collected by centrifugation at 300 × g for 5 min at 4 °C, and then were used for organoid culture or frozen in liquid nitrogen quickly for western blot or quantitative real-time PCR (qRT-PCR).

### Intestinal organoid culture and treatment

The isolated crypts were cultured following the previous report [23]. In brief, 2 ml of DMEM/F12 with 15 mM HEPES (STEMCELL Technologies, Canada) was added to resuspend the isolated crypts, which were transferred to a 15 ml centrifuge tube and centrifuged at 4 °C and 200 × g for 3 min. The crypts were counted under a microscope and a total of 300 crypts were resuspended in 50 µl of Matrigel (CORNING, NY, USA) with a pre-chilled pipette tip and seeded on a 48-well plate. The 48-well plate was transferred into the culture incubator following Matrigel polymerization, 250 µl of IntestiCult™ Organoid Growth Medium (STEMCELL Technologies, Canada) was added to each well, and the crypts were cultured at 37 °C in a humidified air containing 5% CO_2_. The medium was changed every 2 days.

Twenty-four hours following culture, the small molecule selective DR5 agonist Bioymifi (100 nM) [25], non-ATP competitive MEK inhibitor PD0325901 (1 μM) [26] or Wnt3a (100 ng/ml) was added to the medium according to the experimental protocol, and this time point was regarded as 0 h following treatment. The intestinal organoids were observed and photographed under a microscope (Nikon, Tokyo, Japan) at 0 h, 24 h, 48 h and 72 h after treatment, to quantify the organoid area with ImageJ software and count the number of buds. The area of intestinal organoids at 24 h, 48 h and 72 h after treatment was expressed as the ratio compared with the area at 0 h. At least 6 intestinal organoids were randomly assessed for each sample to obtain the mean value of the organoid area and the number of buds per organoid. Intestinal organoids were collected 72 h after treatment for western blot or qRT-PCR.

### TRAIL silencing in organoids

Intestinal organoids were collected with Cell Recovery Solution (CORNING, NY, USA) after 7 days of culture, and digested with 0.25% trypsin-EDTA for 4 min. Then, cells were collected and cultured in 48-well plates with 280 μl of Opti-MEM medium and the transfection mix composed of 1 nmol siRNA (GenePharma, Suzhou, China) and 3 μl of Lipofectamine^TM^3000 (Invitrogen, California, USA) at 37 °C in humidified air containing 5% CO_2_ for 3.5 h. Finally, a total of 4 × 10^5^ cells were re-suspended with 50 µl of Matrigel and cultured in 3D Matrigel with 250 µl of IntestiCult™ Organoid Growth Medium in 48-well plates. After 2 days, the cells were collected for qRT-PCR.

### Western blot analysis

Total protein was collected from ileal tissues, intestinal crypts, or organoids. The protein concentration was determined by a Bicinchoninic Acid Protein Assay kit (Beyotime Institute of Biotechnology, Shanghai, China). Protein samples were separated by sodium dodecyl sulfate–polyacrylamide gel electrophoresis and transferred to polyvinylidene difluoride (PVDF) membranes (Millipore, Massachusetts, USA). The PVDF membranes were blocked with 5% nonfat dry milk and incubated overnight with the primary antibody at 4 °C according to experimental requirements. The primary antibodies are shown in Supplementary Table 3. The next day, the membranes were washed with Tris Buffered Saline with Tween 20 (TBST) and incubated with the corresponding secondary antibodies listed in Supplementary Table 3. Protein bands were visualized using the ChemiDoc XRS system and Image Lab Software following coverage with BeyoECL PLUS (Beyotime Institute of Biotechnology, Shanghai, China), and protein expression was quantified with the ChemiDoc XRS system and Mage Lab Software (Bio-Rad, Hercules, USA).

### mRNA isolation and qRT-PCR

Total RNA of ileal tissues, isolated crypts, or organoids was extracted with SparkZol Reagent (Sparkjade, Shandong, China). Synthesis of cDNA was performed with 2 µg of total RNA using reagents in the PrimeScript^TM^ RT Reagent Kit (Takara, Japan). The PCR reactions were performed with Applied Biosystems (Foster City, CA, USA), and qRT-PCR was finished with an Applied Biosystems StepOne Real-Time PCR System (Thermo Fisher Scientific, USA) using UltraSYBR Mixture (CWBIO, Beijing, China). The relative transcript levels of gene were normalized to β-actin and calculated by the 2^-ΔΔCt^ method. The primers used were listed in Supplementary Table 4.

### RNA sequencing (RNA-seq) of intestinal crypts

Intestinal crypts from wild-type (WT) mice and DR5^-/-^ mice were harvested as delineated in our established protocol and subsequently subjected to RNA-seq. Three individual samples, each derived from a different mouse, were prepared for each group, resulting in a total of six samples. Following extraction, the crypts were preserved in SparkZol Reagent, and then the preserved samples were sent to Genechem Co., Ltd. (Shanghai, China) for RNA sequencing. The high-throughput process converted the sequenced fragments into image data. These images were then translated into clean reads using CASAVA’s base recognition system. The clean reads obtained were aligned to reference transcripts using the HISAT2 software. Quantitative gene expression analysis for each sample was carried out using the feature Counts tool. To identify differentially expressed genes (DEGs), we employed the DESeq2 algorithm. The criteria for selecting DEGs were a |log2(FoldChange)| > 1 and an adjusted *P*-value (padj) < 0.05.

### Drugs and chemicals

Bioymifi was purchased from Selleck (Houston, TX, USA). PD0325901 was purchased from APExBIO (Houston, TX, USA). Wnt3a was purchased from PeproTech (Rocky Hill, NJ, USA).

### Statistical analyses

GraphPad Prism 8.0 was used for statistical analysis. We performed the Shapiro-Wilk test to determine the normality of the data and demonstrated that all data were normally distributed. All data are expressed as the mean ± SEM. Unpaired *t*-tests were employed to compare differences between two groups. One-way ANOVA followed by Tukey’s multiple comparisons test or two-way ANOVA followed by Sidak’s multiple comparisons test was employed to determine significant differences among multiple groups. *P* < 0.05 was considered significant.

## Results

### DR5 gene deletion inhibits small intestinal epithelial function

**Fig. 1:**
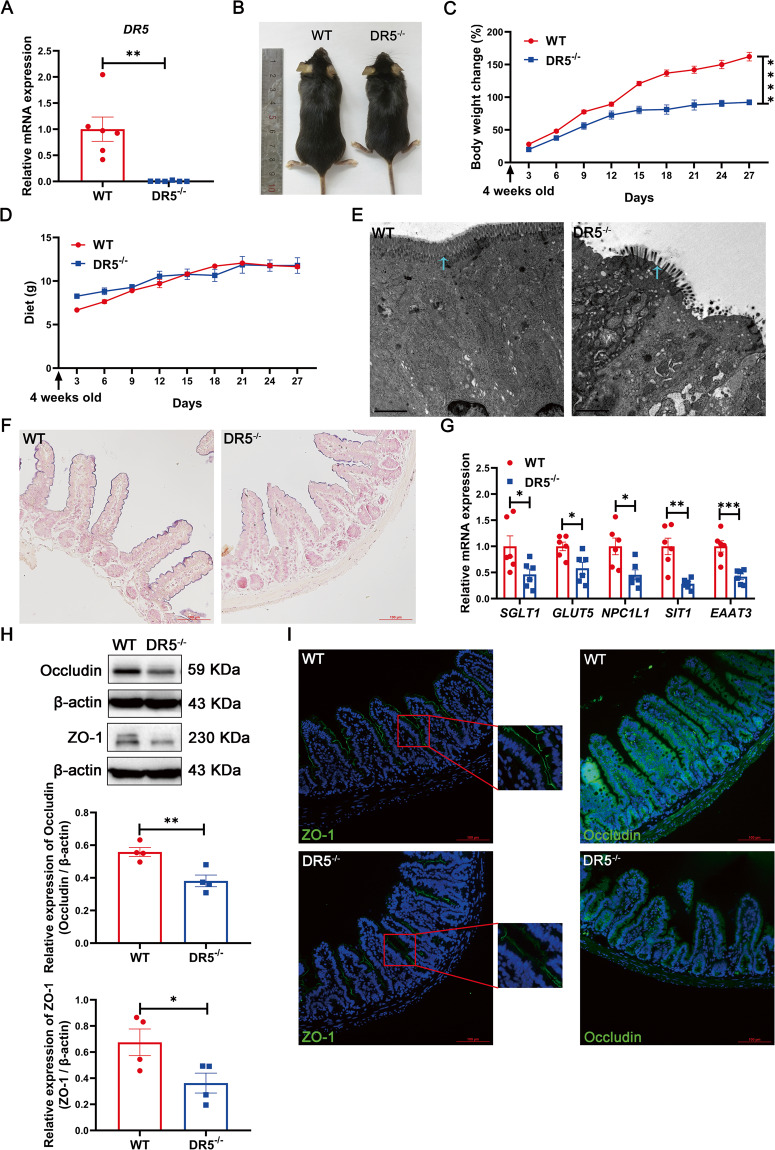
DR5 gene deletion disrupts the intestinal epithelial function. Data were expressed as mean ± SEM. Statistical analyses were performed by the unpaired *t*-tests or two-way ANOVA followed by Sidak’s multiple comparisons test (*P < 0.05, **P < 0.01, ***P < 0.001, ****P < 0.0001). **A** qRT-PCR analysis showed that DR5 gene in ileum was deleted in DR5^-/-^ mice compared with WT mice (*n* = 6). **B** Representative images of DR5^-/-^ mice and WT mice at 8 weeks after birth. **C** The body weight change of DR5^-/-^ mice and WT mice from 4 to 8 weeks after birth. From the 4th week of birth, the weight of the mice was measured every 3 days and expressed as a percentage change from the first measurement (*n* = 10). **D** Food intake of DR5^-/-^ mice and WT mice from 4 to 8 weeks after birth. The food intake of each cage was measured every 3 days and expressed as the average daily food mass per cage per mouse (*n* = 4). **E** Transmission electron microscopy images of ileal epithelial cells in DR5^-/-^ mice and WT mice. The blue arrow indicated the microvilli (scale bars: 2 μm). **F** Images for ALP staining to show the brush border of intestinal epithelial cells (scale bars: 100 μm). **G** qRT-PCR analysis showed the expression of absorption related genes in intestinal epithelial cells of WT and DR5^-/-^ mice (*n* = 6). **H** Representative images and densitometry analysis for the expression of Occludin and ZO-1 in ileal tissue from WT mice and DR5^-/-^ mice (*n* = 4). Both are tight junction proteins that make up the intestinal epithelial barrier. **I** Immunofluorescence staining for ZO-1 and Occludin in ileum of WT mice and DR5^-/-^ mice (scale bars: 100 μm).

DR5^-/-^ mice survived and reproduced normally. As expected, the gene expression of DR5 in the ileum of DR5^-/-^ mice was significantly lower than that of WT mice (Fig. 1A). Despite no difference in daily food intake between the two groups at 4–8 weeks of age, DR5^-/-^ mice were smaller, gained weight more slowly, and had higher faecal dry weight than WT mice (Fig. 1B–D, S1). The microvilli on the apical membrane of intestinal epithelial cells facilitates the absorption of nutrients, and the microvilli of DR5^-/-^ mice were shorter, disordered and sparsely distributed under transmission electron microscopy (Fig. 1E). Compared with that of WT mice, the ileal ALP staining of DR5^-/-^ mice was weaker and discontinuous, suggesting an abnormal apical brush border of epithelial cells (Fig. 1F). Moreover, the expression of absorption-related genes of epithelial cells, including sodium-glucose cotransporter SGLT1, fructose transporter GLUT5, intestinal cholesterol transporter Niemann-Pick C1-like 1 (NPC1L1), imino acid transporter sodium-dependent imino transporter 1 (SIT1), and excitatory amino acid transporter EAAT3 [27–29], was reduced significantly in DR5^-/-^ mice (Fig. 1G). In DR5^-/-^ mice, the expression of the tight junction proteins ZO-1 and Occludin, which constitute the epithelial barrier [30] decreased significantly; ZO-1 was arranged loosely on the outside of the small intestinal villi, and continuity was interrupted or missing; the fluorescence intensity of Occludin decreased (Fig. 1H, I). The weight loss of DR5^-/-^ mice may be related to the dysfunction of epithelial cells.

### Deletion of DR5 led to a decrease in intestinal epithelial cells

**Fig. 2:**
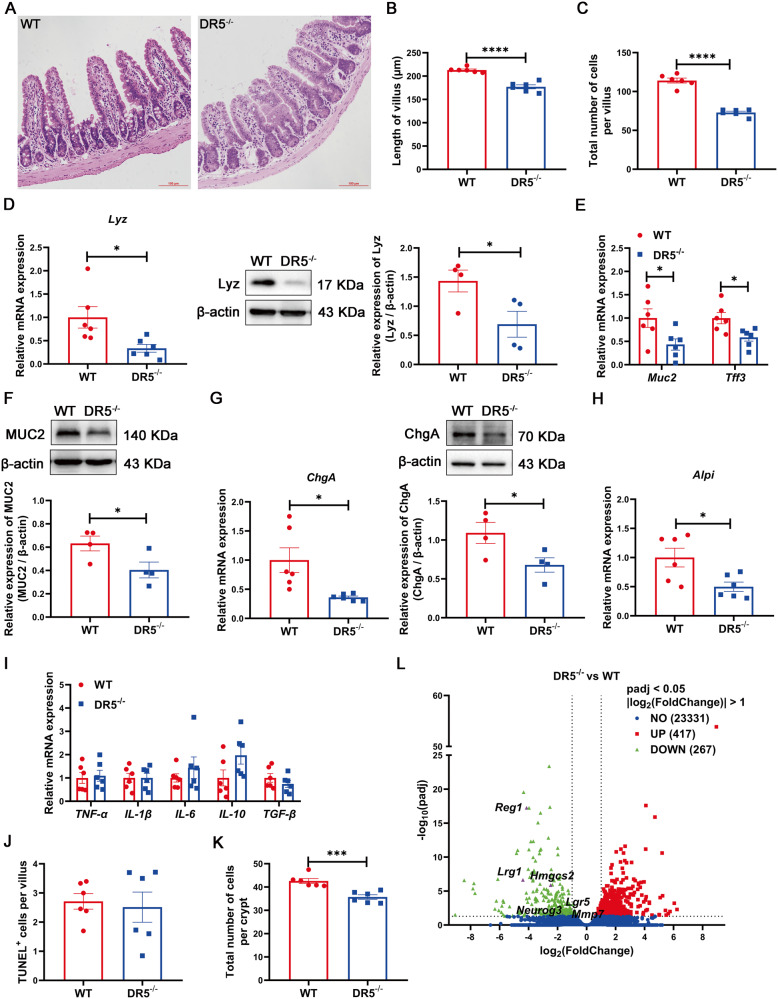
DR5 gene deletion reduces the number of intestinal epithelial cells via targeting ISC. Data were expressed as mean ± SEM. Statistical analyses were performed by the unpaired *t*-tests (*P < 0.05, ***P < 0.001, ****P < 0.0001). **A** Representative images of H&E staining in the ileum of WT mice and DR5^-/-^ mice (scale bars: 100 μm). **B** Quantification of villus length within two groups (*n* = 6). At least 15 correctly aligned villi were counted randomly for each slide and the mean value was calculated. **C** The total number of epithelial cells in ileal villus of WT mice and DR5^-/-^ mice (*n* = 6). At least 15 correctly aligned villi were measured for every slide to get the mean value. **D** qRT-PCR (*n* = 6) and western blot (*n* = 4) were performed to determine the relative mRNA and protein levels of lysozyme (Lyz) in ileum of WT mice and DR5^-/-^ mice. Lyz is the specific marker for Paneth cells. **E** qRT-PCR was performed to determine the relative mRNA levels of Muc2 and Tff3 in ileum of WT mice and DR5^-/-^ mice (*n* = 6). Both are the specific marker genes for goblet cells. **F** Western blot to quantify the protein expression of MUC2 in ileum of WT mice and DR5^-/-^ mice (*n* = 4). **G** qRT-PCR (*n* = 6) and western blot (*n* = 4) were performed to determine the relative mRNA and protein expression of ChgA in ileum of WT mice and DR5^-/-^ mice. It is the specific marker for enteroendocrine cells. **H** qRT-PCR was performed to determine the relative mRNA levels of Alpi in ileum of WT mice and DR5^-/-^ mice (*n* = 6). It is the specific marker for enterocytes. **I** Relative mRNA expression of inflammatory factors in the ileum of WT mice and DR5^-/-^ mice (*n* = 6). **J** Quantification of TUNEL^+^ cells in ileal villus of WT mice and DR5^-/-^ mice (*n* = 6). At least 10 villi were counted randomly for each slide and the mean value was calculated. **K** Total number of epithelial cells in ileal crypts of WT mice and DR5^-/-^ mice (*n* = 6). At least 15 correctly aligned crypts were measured for every slide to get the mean value. **L** The volcano plot visualized the DEGs in ileal crypts from WT mice and DR5^-/-^ mice. Names listed in the figure were DEGs associated with proliferation or differentiation of ISC.

Compared with WT mice, the ileal villi of DR5^-/-^ mice were shorter and the number of differentiated epithelial cells in the villi was reduced (Fig. 2A-C). The gene and/or protein expression of Lyz for Paneth cells, MUC2 and Tff3 for goblet cells, ChgA for enteroendocrine cells and Alpi for enterocytes declined in DR5^-/-^ mice, as did the number of Paneth cells and goblet cells (Fig. 2D–H, S2). The reduction in epithelial cells should be one of the causes of epithelial dysfunction in DR5^-/-^ mice. The decrease in the number of epitheliums might not be caused by the loss of epitheliums, as DR5 deletion did not change the villi structure, the expression of inflammatory factors, and the number of TUNEL^+^ cells in the villi (Fig. 2I, J, S3). DR5^-/-^ mice had significantly reduced numbers of epithelial cells in crypts (Fig. 2K), where accommodate ISCs, TA cells, and Paneth cells, which are critical for epithelial renewal. Therefore, we performed RNA-seq with ileal crypts and found 267 genes were down-regulated due to DR5 deletion, including the ISC marker gene Lgr5, and the ISC differentiation determining genes Matrix metallopeptidase 7 (Mmp7), Neurog3 (Ngn3), Lrg1, Reg1 and Hydroxymethylglutaryl CoA synthase 2 (Hmgcs2) (Fig. 2L). We suspect that the decrease in epithelial cells is caused by altered ISC activity, leading to epithelial dysfunction.

### DR5 deletion results in impaired proliferation and differentiation of ISC

**Fig. 3:**
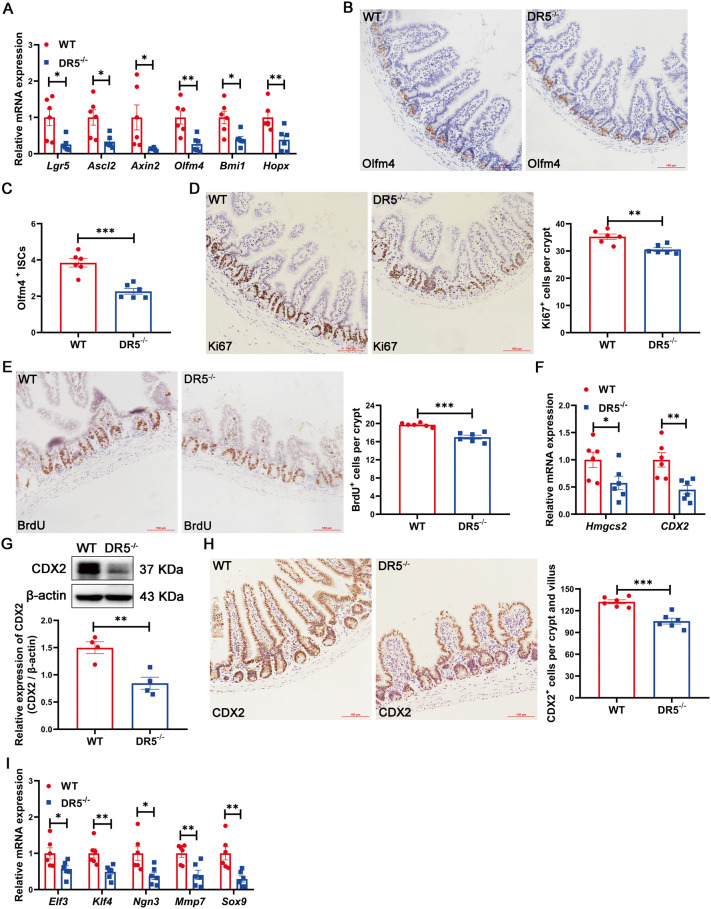
DR5 is needed for the proliferation and differentiation of ISC in the ileum. Data were expressed as mean ± SEM. Statistical analyses were performed by the unpaired *t*-tests (*P < 0.05, **P < 0.01, ***P < 0.001). **A** qRT-PCR analysis to show the expression of ISC marker genes in ileum of WT and DR5^-/-^ mice (*n* = 6). **B** Representative images for immunohistochemical staining of Olfm4 within two groups (scale bars: 100 μm). **C** Statistical graph of the Olfm4^+^ ISCs number in WT mice and DR5^-/-^ mice (*n* = 6). At least 15 crypts were counted randomly for each slide and the mean value was calculated. **D** Representative images for immunohistochemical staining of Ki67 (scale bars: 100 μm) and quantification of Ki67^+^ cells in ileal crypt in WT mice and DR5^-/-^ mice (*n* = 6). At least 15 crypts were counted randomly for each slide and the mean value was calculated. **E** Representative images for immunohistochemical staining of BrdU (scale bars: 100 μm) and quantification of BrdU^+^ cells in ileal crypt in WT mice and DR5^-/-^ mice (*n* = 6). At least 15 crypts were counted randomly for each slide and the mean value was calculated. **F** qRT-PCR analysis of Hmgcs2 and CDX2 in ileum of WT mice and DR5^-/-^ mice (*n* = 6). **G** The protein expression of CDX2 in ileum of WT mice and DR5^-/-^ mice (*n* = 4). **H** Representative images for immunohistochemical staining of CDX2 (scale bars: 100 μm) and quantification of CDX2^+^ cells in ileum of WT mice and DR5^-/-^ mice (*n* = 6). At least 15 correctly aligned villi including crypts were counted randomly for each slide and the mean value was calculated. **I** qRT-PCR was performed to examine the expression of ISC lineage differentiation genes in ileum of WT mice and DR5^-/-^ mice (*n* = 6). Elf3 for enterocyte fate specification, Klf4 for goblet cell fate specification, Ngn3 for enteroendocrine cell fate specification, Mmp7 and Sox9 for Paneth cells fate specification were detected in the ileum.

DR5 loss lowered the expression of active ISC marker genes (Lgr5, Ascl2, Axin2, Olfm4) and +4 ISC marker genes (Bmi1 and Hopx) [3] in ileum, along with the number of Olfm4^+^ ISCs (Fig. 3A–C). The number of Ki67^+^ cells (proliferating cells) and BrdU^+^ cells (S phase proliferating cells) [31, 32] in crypts decreased significantly (Fig. 3D, E). Both suggested that ISC proliferation was inhibited. For ISC differentiation, the gene expression of Hmgcs2 and Caudal-related homeobox transcription factor 2 (CDX2), as well as CDX2 protein and CDX2^+^ cells declined in the ileum of DR5^-/-^ mice (Fig. 3F–H). Hmgcs2 and CDX2 are genes promoting ISC differentiation [33, 34]. The ISC lineage differentiation genes, including E47-like factor 3 (Elf3) for enterocyte fate, Krüppel-like factor 4 (Klf4) for goblet cell fate, Ngn3 for enteroendocrine cell fate, and Mmp7 and Sry-related HMG box 9 (Sox9) for Paneth cell fate [4] were down-regulated in DR5^-/-^ mice (Fig. 3I). Overall, we conclude that DR5 is needed for ISC activity.

### DR5 expression in crypts is involved in the regulation of ISC activity

**Fig. 4:**
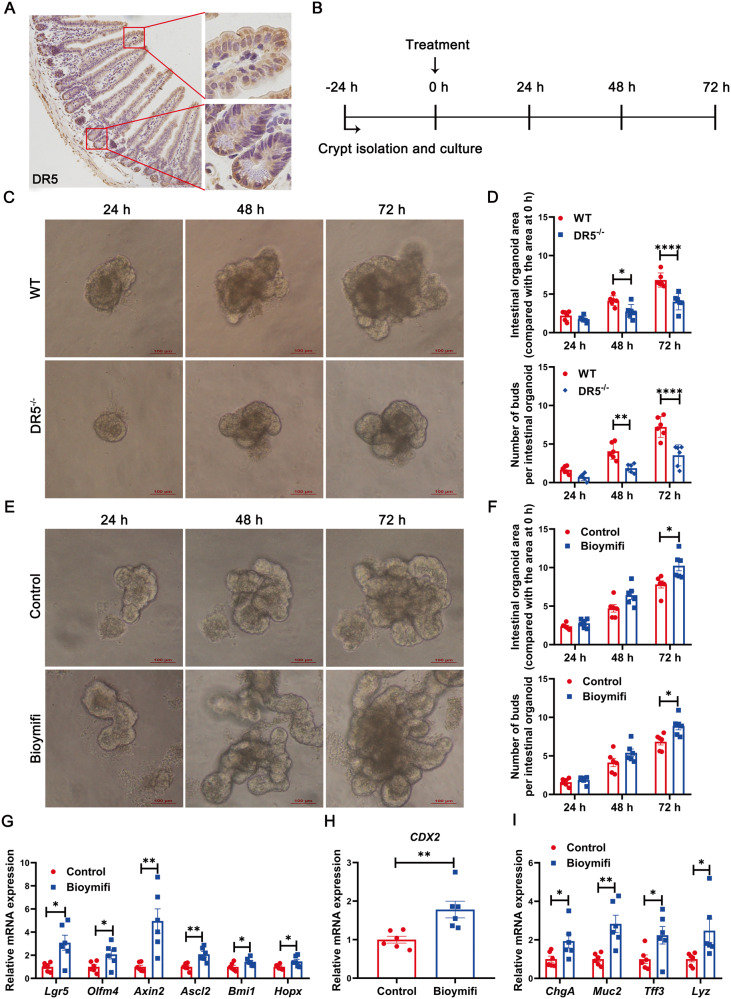
DR5 expression in intestinal crypts is involved in the regulation of ISC activity. Data were expressed as mean ± SEM. Statistical analyses were performed by the unpaired *t*-tests or two-way ANOVA followed by Sidak’s multiple comparisons test (*P < 0.05, **P < 0.01, ****P < 0.0001). **A** Immunohistochemical staining of DR5 in ileum of WT mice (scale bar: 100 μm). **B** The time axis for organoid culture and treatment. Ileal crypts were isolated and cultured in the Intesticult™ Organoid Growth Medium. 24 h after crypts were inoculated the organoids were treated with the drug according to the experimental protocol. **C** Representative images of intestinal organoids growth generated from ileal crypts of WT mice and DR5^-/-^ mice (scale bars: 100 μm). **D** Quantification of the organoids area, the number of buds derived from crypts of WT mice and DR5^-/-^ mice (*n* = 6). The area of intestinal organoids 24, 48 and 72 h after treatment were evaluated as the ratio compared with the area at 0 h. At least 6 intestinal organoids were randomly detected for each sample to get the mean value. For the number of buds, at least 6 intestinal organoids were randomly detected in each sample to obtain the average number of buds per organoid at 24, 48 and 72 h after treatment. **E** Representative images of intestinal organoids generated from ileal crypts of WT mice treated with or without Bioymifi (100 nM) (scale bars: 100 μm). **F** Quantitative analysis of the area, number of buds of in Bioymifi (100 nM) treatment and untreated organoids derived from crypts of WT mice (*n* = 6). Organoid area and budding were quantified according to the method shown in Fig. 4D. **G** qRT-PCR analysis of ISC marker genes expression in Bioymifi (100 nM) treated and untreated organoids derived from crypts of WT mice (*n* = 6). The gene expression was tested 72 h following Bioymifi treatment. **H** qRT-PCR analysis of CDX2 expression in Bioymifi (100 nM) treated and untreated organoids derived from crypts of WT mice (*n* = 6). The CDX2 expression was tested 72 h following Bioymifi treatment. **I** qRT-PCR analysis of epithelial cells marker genes expression in Bioymifi (100 nM) treated and untreated organoids derived from crypts of WT mice (*n* = 6). ChgA for enteroendocrine cell, Muc2 and Tff3 for goblet cell and Lyz for Paneth cell. All the genes were tested 72 h following Bioymifi treatment.

DR5 expressing in the nucleus was involved in cell proliferation and differentiation [35]. DR5 located not only in the membrane, cytoplasm, but also in the nucleus in intestinal crypts (Fig. 4A). DR5 expression in crypts might be involved in the regulation of ISC activity. To test this hypothesis, we performed organoid culture to determine the role of DR5 in ISC activity and epithelial regeneration (Fig. 4B) [23].

The growth of DR5^-/-^ crypts-derived organoids was inhibited, organoid area and bud number decreased, while treatment with the selective DR5 agonist Bioymifi for 72 h promoted the increase of organoid buds and area (Fig. 4C–F), indicating DR5 in crypts was involved in the regulation of epithelial regeneration. Consistent with these findings, the mRNA expression of ISC marker genes Lgr5, Ascl2, Axin2, Olfm4, Bmi1, and Hopx, the ISC differentiation determining gene CDX2, and the epithelial cell marker genes ChgA, Muc2, Tff3, and Lyz was increased following Bioymifi administration (Fig. 4G–I), suggesting enhanced epithelial regeneration was due to ISC activation. Bioymifi had no effect on organoids derived from DR5^-/-^ crypts, while reducing TRAIL expression in organoids derived from WT crypts inhibited the mRNA expression of Lgr5, CDX2, Lyz and Muc2 (Fig. S4–S5). Data from organoids demonstrate that DR5 expression in crypts regulates ISC activity in a ligand-dependent manner, and then affects epithelial regeneration.

### DR5 deficiency results in apoptosis of Paneth cells and TA cells

**Fig. 5:**
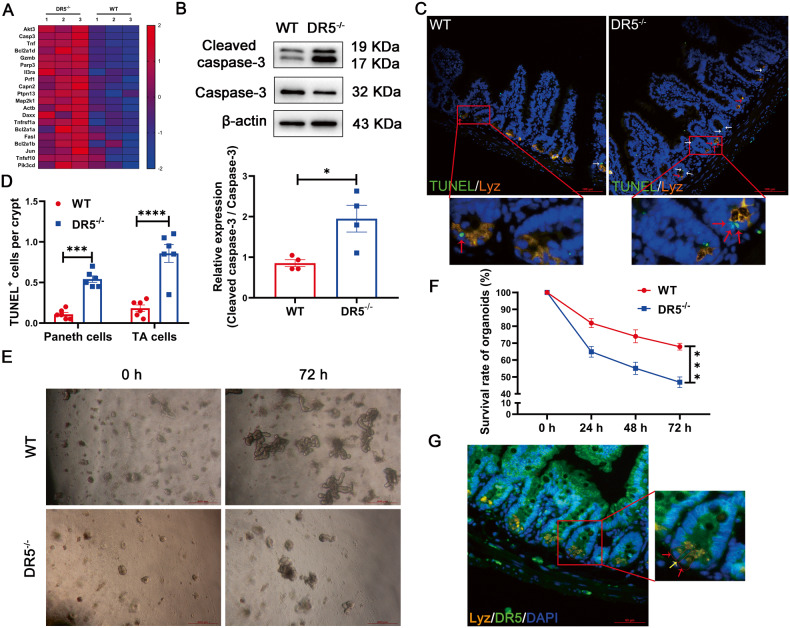
DR5 loss induces excessive apoptosis of Paneth cells and TA cells. Data were expressed as mean ± SEM. Statistical analyses were performed by the unpaired *t*-tests or two-way ANOVA followed by Sidak’s multiple comparisons test (*P < 0.05, ***P < 0.001, ****P < 0.0001). **A** The analysis of KEGG apoptosis signalling pathway gene set based on RNA-seq. The top 20 genes are listed in the heatmap, which shows that the expression of apoptosis related genes is upregulated in intestinal crypts of DR5^-/-^ mice compared with that of WT mice (*n* = 3). In this scheme, deep red corresponds to heightened gene expression levels, while light blue denotes lower expression. **B** Western blot was performed to quantify the cleaved caspase-3 protein expression in ileal crypts from WT mice and DR5^-/-^ mice (*n* = 4). **C** Representative pictures of staining of Lyz, TUNEL, and DAPI (nuclei) in ileum of WT mice and DR5^-/-^ mice (scale bars: 100 μm). The red arrows indicate TUENL^+^ Paneth cells, and the white arrows indicate the TUNEL^+^ TA cells. **D** Statistical analysis to show the difference of the TUENL^+^ Paneth cells and TUNEL^+^ TA cells between WT mice and DR5^-/-^ mice (*n* = 6). At least 20 crypts were counted randomly for each slide and the mean value was calculated. **E** Representative images showing organoids derived from crypts of WT and DR5^-/-^ mice 72 h after culture (scale bars: 500 μm). **F** Dynamic changes of organoid survival in WT and DR5^-/-^ mice (*n* = 6). For each sample, four 40-fold visual fields were randomly selected to count the number of organoids under each visual field, and the average value was obtained. The number of organoids at different time points was normalized with the number of organoids at 0 h to reflect the organoid survival rate. **G** Immunofluorescence co-labelled Lyz and DR5 in ileum. The red arrows indicate the DR5^+^ Paneth cells and the yellow arrow indicates DR5^+^ ISC in the basement of crypt (scale bar: 50 μm).

Based on RNA-seq, we analyzed the KEGG apoptosis pathway gene set and listed the top 20 genes. The heatmap showed elevated expression of apoptosis-related genes within the crypts of DR5^-/-^ mice compared to WT mice (Fig. 5A). This trend was further validated by a higher expression of cleaved caspase-3, a higher number of TUNEL^+^ Paneth cells and TUNEL^+^ TA cells in DR5^-/-^ mice’s crypts than WT mice’s, suggesting increased apoptosis in Paneth cells and TA cells (Fig. 5B–D). Consistent with enhanced apoptosis, organoids derived from DR5^-/-^ crypts exhibited a reduced survival rate compared to those from WT crypts (Fig. 5E, F). DR5 expression was evident in ISCs, Paneth cells, and TA cells (Fig. 5G). Given that Paneth cells function as ISC niche cells and TA cells serve as swiftly dividing lineage-committed progenitor cells [8, 36], any dysfunction in these cells can impede epithelial renewal in DR5^-/-^ mice.

### The effect of DR5 on ISC activity is partly related to the production of Paneth cell-derived ISC niche factors

**Fig. 6:**
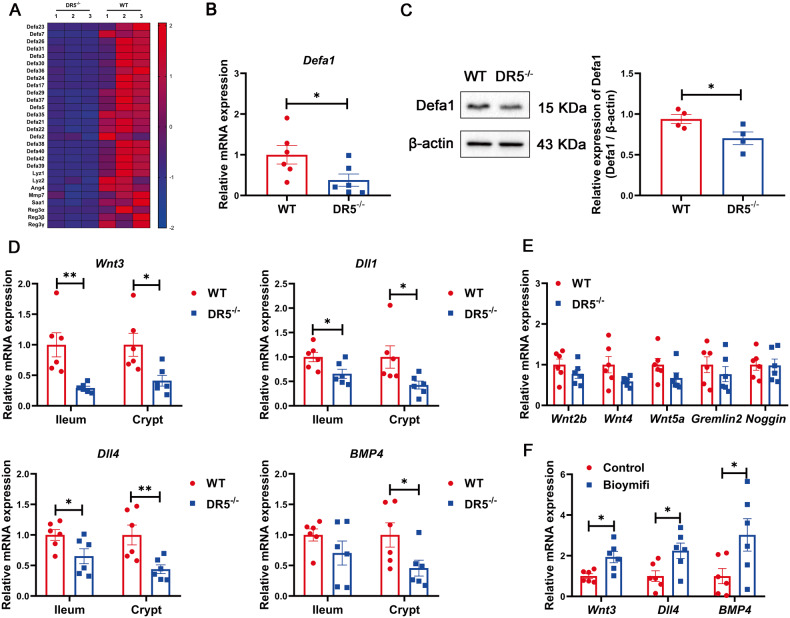
DR5 loss impairs the production of Paneth cell-derived ISC niche factors. Data were expressed as mean ± SEM. Statistical analyses were performed by the unpaired *t*-tests (*P < 0.05, **P < 0.01). **A** Heatmap showing the expression of antibacterial peptide related genes within DEGs based on RNA-seq data of crypts (*n* = 3). The heatmap used a gradient from light blue (lower expression) to deep red (heightened expression) to represent gene expression levels. **B** qRT-PCR analysis of Defensin alpha 1 (Defa1) gene expression in ileum of WT mice and DR5^-/-^ mice (*n* = 6). **C** Protein expression of Defa1 in ileum of WT mice and DR5^-/-^ mice (*n* = 4). **D** qRT-PCR was performed to determine the relative mRNA level of Wnt3, Dll1, Dll4 and BMP4 in ileal tissue and ileal crypt (*n* = 6). **E** qRT-PCR analysis of Wnt ligands Wnt2b, Wnt4 and Wnt5a, BMP signalling antagonist factors Gremlin2 and Noggin in ileal tissue from WT mice and DR5^-/-^ mice (*n* = 6). They are all nonepithelial derived ISC niche factors. **F** qRT-PCR was performed to examine the effect of Bioymifi (100 nM) administration in vitro on Wnt3, Dll4 and BMP4 gene expression in organoids derived from crypts of WT mice (*n* = 6).

Paneth cells are heavily granulated epithelial cells that secrete antimicrobial peptides (AMPs) and ISCs niche factors [37, 38]. We identified Paneth cells AMP-related genes among DEGs from the sequencing results, and constructed a heatmap, which showed decreased expression of AMP-related genes within crypts after DR5 deletion (Fig. 6A). For validation, the expression of Lyz and Defa1 decreased at the gene and protein levels in DR5^-/-^ mice (Figs. 2D, 6B, C). The ISC niche factors Wnt3, Dll1 and Dll4 [38, 39] in both the ileum and crypts of DR5^-/-^ mice were significantly lower than those in WT mice, while DR5 deletion reduced only the gene expression of BMP4 in crypts (Fig. 6D). Nonepithelial origin ISC niche factors in the ileum of DR5^-/-^ mice, including Wnt2b, Wnt4, Wnt5a, Gremlin2 and Noggin, were the same as those in WT mice (Fig. 6E). Bioymifi administration upregulated the mRNA expression of Wnt3, Dll4 and BMP4 in organoids (Fig. 6F). Therefore, DR5 is involved in the production of Paneth cell-derived ISC niche factors.

**Fig. 7:**
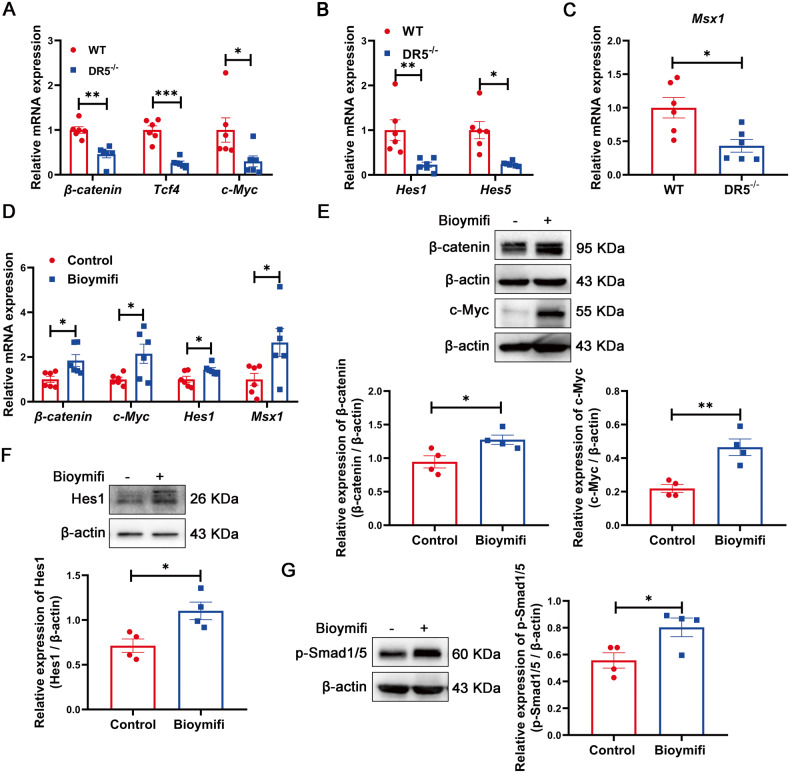
DR5 is involved in the regulation of Wnt, Notch and BMP pathway in vivo and in ex vivo organoids. Data were expressed as mean ± SEM. Statistical analyses were performed by the unpaired *t*-tests (*P < 0.05, **P < 0.01, ***P < 0.001). **A** qRT-PCR analysis of β-catenin, Tcf4 and c-Myc in ileal crypts of WT mice and DR5^-/-^ mice (*n* = 6). They are Wnt signalling target genes. **B** qRT-PCR analysis of Hes1 and Hes5 in ileal crypts of WT mice and DR5^-/-^ mice (*n* = 6). They are Notch signalling target genes. **C** qRT-PCR analysis of Msx1 in ileal crypts of WT mice and DR5^-/-^ mice (*n* = 6). It is the BMP signalling target gene. **D** Effect of Bioymifi (100 nM) administration in vitro on the mRNA expression of β-catenin, c-Myc, Hes1 and Msx1 in organoids (*n* = 6). **E** Effect of Bioymifi (100 nM) administration in vitro on the protein expression of β-catenin and c-Myc in organoids (*n* = 4). **F** Effect of Bioymifi (100 nM) administration in vitro on the protein expression of Hes1 in organoids (*n* = 4). **G** Effect of Bioymifi (100 nM) administration in vitro on the protein expression of p-Smad1/5 in organoids (*n* = 4).

Next, we tested whether reductions in Wnt3, Dll1, Dll4, and BMP4 affected the activity of Wnt, Notch, and BMP signalling, which are all recognized ISC regulatory pathways [40]. Compared with those of WT mice, the core effector of Wnt signalling, β-catenin, Tcf4 and its target gene c-Myc were down-regulated in crypts of DR5^-/-^ mice (Fig. 7A), as well as the Notch signalling target genes Hes1 and Hes5 [41], BMP signalling target gene Msx1[42] (Fig. 7B, C). In organoids, Bioymifi upregulated the gene expression of β-catenin, c-Myc, Hes1 and Msx1, the protein expression of β-catenin, c-Myc, Hes1 and the effector protein of BMP pathway, p-Smad1/5 (Fig. 7D–G). Wnt3 secreted by Paneth cells is the only Wnt ligand in organoid, which is crucial to the organoid formation [43]. Wnt3a supplementation improved DR5^-/-^ organoid growth but could not restore it to normal (Fig. S6).

We, therefore, favour the conclusion that the role of DR5 on ISC activity is at least partially related to the production of Paneth cell-derived ISC niche factors.

### DR5 regulates ISC activity and epithelial cell renewal depending on ERK1/2 activity

**Fig. 8:**
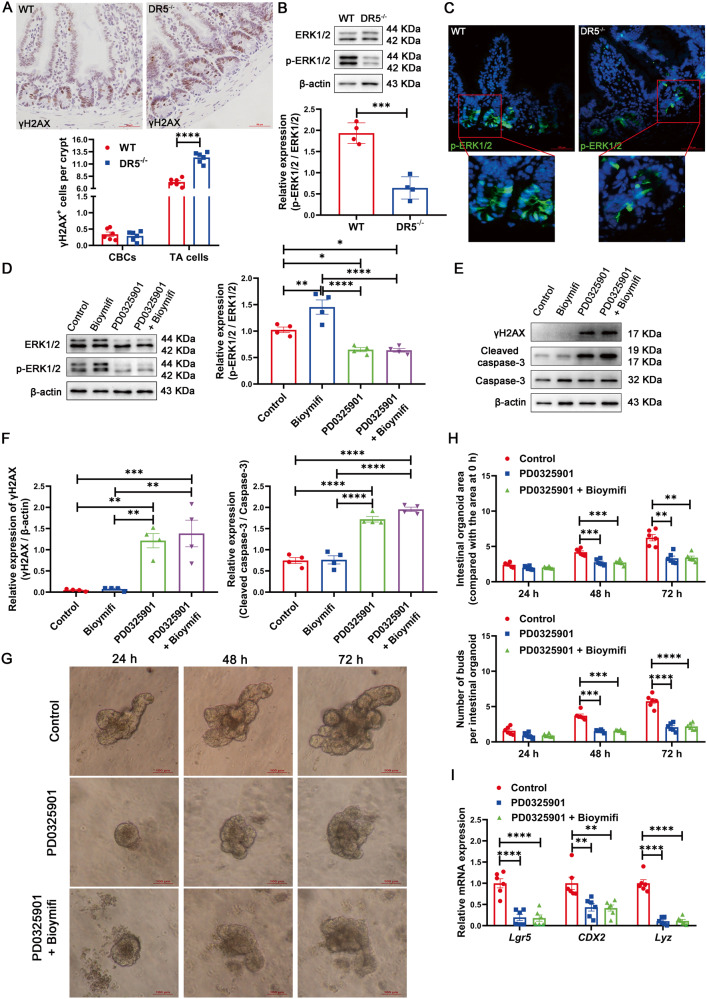
DR5 regulates ISC activity and epithelial cell renewal depending on ERK1/2 activity. Data were expressed as mean ± SEM. Statistical analyses were performed by the unpaired *t*-tests or one-way ANOVA followed by Tukey’s multiple comparisons test or two-way ANOVA followed by Sidak’s multiple comparisons test (*P < 0.05, **P < 0.01, ***P < 0.001, ****P < 0.0001). **A** Representative immunohistochemical images for γH2AX in ileum of WT mice and DR5^-/-^ mice (scale bars: 50 μm) and the statistical graph of γH2AX^+^ cells in CBC cells and TA cells within two groups (*n* = 6). At least 15 crypts were counted randomly for each slide and the mean value was calculated. **B** Representative western blots photographs for p-ERK1/2 expression in the crypts of WT mice and DR5^-/-^ mice and the statistical analysis of ERK1/2 activity within two groups (*n* = 4). **C** Immunofluorescence images for p-ERK1/2 staining in ileum of WT mice and DR5^-/-^ mice (scale bars: 100 μm). **D** Representative photographs and statistical analysis to show the role of PD0325901 (1 μM) on ERK1/2 activation and its effect on Bioymifi (100 nM) activating ERK1/2 in organoids (*n* = 4). 72 h after Bioymifi (100 nM) administration in the presence or absence of PD0325901 (1 μM) in organoids, the organoids were collected to examine the p-ERK1/2 expression. **E** Representative western blot photographs to show the effect of PD0325901 (1 μM) and Bioymifi (100 nM) on γH2AX and cleaved caspase-3 expression in organoids, and the role of PD0325901 on Bioymifi’s effect. The protein expression was examined 72 h following treatment. **F** Quantify the Western blot results to show the effect of PD0325901 and Bioymifi on γH2AX and cleaved caspase-3 expression in organoids (*n* = 4). **G** Representative images to show the role of PD0325901 (1 μM) on organoids formation and its effect on Bioymifi (100 nM) prompting organoids growth (scale bars: 100 μm). **H** Statistical analysis of organoids area and buds to show the effect of PD0325901 on organoids growth and on Bioymifi prompting organoids growth (*n* = 6). Organoid area and budding were quantified according to the method shown in Fig. 4D. **I** The effect of PD0325901 (1 μM) on the Lgr5, CDX2 and Lyz gene expression in organoids and its effect on the up-regulation of Lgr5, CDX2 and Lyz gene expression induced by Bioymifi (100 nM) (*n* = 6).

γH2AX is a sensitive biomarker for DNA damage [44]. Consistent with the increased apoptosis of TA cells, the number of γH2AX^+^ TA cells was increased after DR5 deletion (Fig. 8A). p-ERK1/2 is expressed in intestinal crypts, and the inhibition of p-ERK1/2 activity is related to DNA damage in crypts [45]. The crypts of DR5^-/-^ mice had reduced expression of p-ERK1/2, including in Paneth cells, ISCs and TA cells (Fig. 8B, C). Treatment with the MEK inhibitor PD0325901 for 72 h inhibited the activation of ERK1/2 in organoids and eliminated the effect of Bioymifi in activating ERK1/2 (Fig. 8D ). Along with inhibition of ERK1/2 activity induced by PD0325901, the protein expression of γH2AX and cleaved caspase-3 increased, the organoid growth and gene expression of Lgr5, CDX2, and Lyz were inhibited (Fig. 8E–I). Importantly, PD0325901 completely eliminated the effect of Bioymifi on the growth of WT crypts-derived organoids and the gene expression of Lgr5, CDX2, and Lyz, but had no effect on the growth of DR5^-/-^ crypts-derived organoids (Fig. 8G-I, S7). Bioymifi alone did not change the expression of γH2AX and cleaved caspase-3 in organoids (Fig. 8E, F ). Thus, DR5 regulates ISC activity and epithelial cell renewal in a manner relying on ERK1/2 activity in crypts.

## Discussion

Our study confirmed that DR5 expressed in ileal crypts is needed for ISC activity during epithelial renewal. DR5 deficiency reduced epithelial cell turnover by inhibiting ISC proliferation and differentiation, resulting in impaired epithelial cell function. The role of DR5 in ISC activity was achieved by influencing the production of Paneth cell-derived ISC niche factors and ERK 1/2 activity in crypts.

Although food intake did not change, DR5^-/-^ mice showed delayed weight gain, which might be due to disruption of epithelial absorption function. There may be multiple reasons why DR5 loss leaded to dysfunction of intestinal epithelial cells, but there is no doubt that the overall reduction in differentiated epithelial cells may be one of causes for this change. The intestinal epithelium is the most rapidly renewed tissue in adult mammals and its homoeostasis depends on the balance between epithelial renewal and loss [7, 46]. There was no evidence that DR5 knockout caused excessive loss of intestinal epithelial cells, while DR5^-/-^ mice had a reduced number of proliferating cells in crypts, and RNA-seq of crypts showed that DR5 deletion resulted in down-regulation of Lgr5, Hmgcs2, Mmp7 and Ngn3. Lgr5 is one of the most reliable active ISCs markers, and Lgr5^+^ ISCs can generate all epithelial lineages [5]. Hmgcs2 is a mitochondrial ketone body synthetase that promotes ISC differentiation by regulating the synthesis of β-hydroxybutyric acid and acetoacetic acid [33]. Mmp7 and Ngn3 specify the differentiation and maturation of Paneth cells and enteroendocrine cells, respectively [4]. Further validation experiments confirmed the reliability of RNA-seq. The expression of ISC marker genes, the number of ISCs and proliferating cells in crypts were significantly decreased in DR5^-/-^ mice, reflecting the inhibition of the proliferation ability of ISCs and TA cells. For ISC differentiation, DR5 gene loss decreased the gene expression of Hmgcs2 and CDX2, CDX2 protein and CDX2^+^ cells in the ileum. The homologous protein encoded by CDX2 gene is critical for intestinal integrity and epithelial differentiation [34]. In particular, four sets of lineage signature genes, Elf3 (enterocytes), Klf4(goblet cells), Ngn3(enteroendocrine cells), Mmp7 and Sox9 (Paneth cells) [4] were decreased in DR5^-/-^ mice. Therefore, DR5 is involved in the maintenance of ISC activity, which is crucial for epithelial cell renewal [7, 47].

To identify the mechanism by which DR5 regulated ISC activity, we established an epithelial model [23]. In this model, isolated crypts formed “intestinal organoids” including all epithelial cell types in the 3D culture system. Epithelial regeneration was significantly inhibited in organoids from DR5^-/-^ crypts compared to WT’s organoids. Bioymifi promoted the formation of intestinal organoids from WT’s crypts, and the expression of ISC marker genes, differentiation genes and epithelial cell marker genes. Bioymifi exerted its role via activating DR5, since it had no effect on DR5^-/-^ organoids. In addition, endogenous DR5 ligand, i.e TRAIL silences in organoids inhibited the expression of Lgr5, Lyz, and Muc2. Clearly, data from organoids demonstrates that DR5 expressed in crypts regulates ISC activity in a ligand-dependent manner, thereby affecting epithelial regeneration. KEGG pathway analysis, and the increased expression of cleaved caspase-3 in crypts of DR5^-/-^ mice revealed the excessive apoptosis of crypts, which resulted in a lower survival rate of DR5^-/-^ organoids. Increased apoptosis occurs in Paneth cells and TA cells. Given that TA cells and Paneth cells both express DR5 and play key role in epithelial cell renewal [4, 6], they are highly likely to be the target cells of DR5 action.

Paneth cells secrete ISC niche factors, such as Wnt3, Dll1 and Dll4 to support neighbouring ISCs [38]. The expression of Wnt3, Dll1 and Dll4 both in ileal segment and crypts was significantly decreased in DR5^-/-^ mice, while BMP pathway ligand BMP4 decreased only in crypts. Paneth cells are the key sources of Wnt3, Dll1 and Dll4, but, BMPs are mainly derived from nonepithelial cell components, not Paneth cells [9, 48]. The different change of BMP4 suggested that DR5 might only affect Paneth cell-derived ISC niche factors. The normal expression of nonepithelial derived ISC niche factors in DR5^-/-^ mice and the up-regulation of Wnt3, Dll4 and BMP4 in Bioymifi-treated organoids, where only epithelial-derived ISC niche factors existed [23], confirmed this speculation. The reduction in ISC niche factors might be related to apoptosis of Paneth cells due to DR5 loss, which needs to be further clarified in the future.

Wnt3, Dll1, Dll4, and BMP4 activate Wnt, Notch and BMP signalling in ISC, respectively, to regulate ISC activity. Consistent with the decrease in Wnt3, Dll1, Dll4 and BMP4 from Paneth cells, the key downstream efforts and/or target genes of Wnt, Notch and BMP signalling were decreased significantly in crypts of DR5^-/-^ mice. Accordingly, Bioymifi activated Wnt, Notch and BMP pathway in organoids. Wnt, Notch, and BMP pathways are closely coordinated to precisely control ISC activity [4]. The ISC marker genes Lgr5, Axin2 and Ascl2 are Wnt signalling target genes, and Olfm4 is Notch signalling target gene [41]. Activation of Wnt and Notch signalling promotes the proliferation of ISC, while BMP signalling negatively regulates ISC proliferation [39, 48, 49]. In terms of ISC differentiation, Notch signalling activation promotes enterocyte differentiation and inhibits secretory cell fate, while Wnt signalling promotes the differentiation of ISC into secretory cell [4, 9]. Undoubtedly, changes in Wnt, Notch and BMP signalling caused by DR5 deletion can affect the ISC activity, while it is unclear which signalling plays a decisive role in this process.

Paneth cells are essential for organoid growth, but their role can be completely replaced by Wnt3a supplementation [50]. However, Wnt3a supplementation improved but did not completely reverse the growth inhibition of DR5^-/-^ crypts-derived organoids. Previous reports showed that total loss of Paneth cells did not change ISC proliferation in vivo because nonepithelial niche cells compensated for the role of Paneth cells [23, 50]. Differently, DR5^-/-^ mice showed obvious inhibition of ISC activity. Therefore, the reduction of Paneth cell-derived ISCs niche factors in DR5^-/-^ mice is only one mechanism for ISC inhibition, and there should be another mechanism involved in the regulation of ISC activity by DR5.

In addition to Paneth cells, the apoptosis of TA cells also increased in DR5^-/-^mice. The quick proliferation of TA cells is crucial for intestinal epithelial homoeostasis [6]. Rapidly proliferating cells have a higher probability of DNA damage than other cells, which increases the probability of apoptosis [51, 52]. Consistent with this, the increased γH2AX^+^ TA cells of DR5^-/-^ mice suggested enhanced DNA damage [44]. Activation of ERK1/2 could protect against DNA damage [53–56] and we reported that inhibition of ERK1/2 activity was involved in DNA damage and apoptosis of intestinal crypts [45]. Consistent with the literatures [45, 53–56], here we found inhibiting the ERK1/2 activity in organoids with PD0325901 promoted the expression of γH2AX and cleaved caspase-3. Overactivated EGFR-ERK1/2 signalling drive intestinal epithelial renewal by promoting ISC proliferation and differentiation in porcine intestinal organoids [57, 58], while inhibiting ERK1/2 attenuates the proliferation of ISCs and their differentiation into TA cells and secretory cells [59, 60]. Similarly, PD0325901 dampened organoid formation and the expression of Lgr5, CDX2 and Lyz. DR5 activation induced the proliferation of naive fibroblasts by activating ERK1/2 [61]. Therefore, we hypothesized that DR5 regulated ISC activity via ERK1/2 pathway. DR5 loss did inhibit the ERK1/2 activity in ileal crypts in vivo, whereas DR5 activation with Bioymifi stimulated ERK1/2 in organoids. Importantly, once the effect of Bioymifi on ERK1/2 activity was blocked by PD0325901, its effects on organoid growth and Lgr5, CDX2 and Lyz gene expression were restricted. In addition, PD0325901 alone did not affect the formation of DR5^-/-^ crypts-derived organoids. Therefore, the effects of DR5 on ISC activity and epithelial regeneration depend on its role in ERK1/2 activity. Finally, we noted that Bioymifi alone exerted no effect on the expression of γH2AX and cleaved caspase-3 in organoids. We determined that the physiological activity of ERK1/2 was sufficient to protect intestinal crypts from DNA damage and apoptosis, so Bioymifi-induced overactivation of ERK1/2 exerted no further effect.

In summary, DR5 expression in intestinal crypts is necessary for ISC activity during epithelial renewal. DR5 regulates the ISC activity by influencing the production of Paneth cell-derived ISC niche factors and maintaining ERK1/2 activity in intestinal crypts. This study confirms a new mechanism of intestinal epithelial renewal at homoeostasis.

### Supplementary information

Supplementary figure legends

Figure S1

Fgiure S2

Fgiure S3

Fgiure S4

Figure S5

Figure S6

Figure S7

Original data

Supplementary Table 1

Supplementary Table 2

Supplementary Table 3

Supplementary Table 4

reproducibility checklist

## Data Availability

Data are available from the corresponding author on reasonable request.
