# Supplementary material for: Death receptor 5 is required for intestinal stem cell activity during intestinal epithelial renewal at homoeostasis
Source: Cell Death Dis. 2024 Jan 10;15(1):27. doi: 10.1038/s41419-023-06409-4 (PMC10782029; doi:10.1038/s41419-023-06409-4)
Supplement: Supplementary file 1 — Supplementary figure legends [file 41419_2023_6409_MOESM1_ESM.docx]

**Supplementary Figure Legends**

**Figure S1 DR5 deletion increases the faecal dry weight**

Data were expressed as mean ± SEM. Statistical analyses were performed by the unpaired t-tests (****P* < 0.001, n=6). Faeces of 8-week-old mice within 30 min was collected at 9 am to examine the dry weight of feces. The measurement was performed for 3 consecutive days, and the mean value was taken.

**Figure S2 DR5 deletion results in a decrease in the number of Paneth cells and goblet cells**

Data were expressed as mean ± SEM. Statistical analyses were performed by the unpaired t-tests (**P* < 0.05, ***P* < 0.01, ****P* < 0.001).

A. Immunohistochemical staining of Lyz (scale bars: 100 μm) and statistical graph of the number of Lyz^+^ Paneth cells in the crypt of WT mice and DR5^-/-^ mice (n=6). At least 15 crypts were counted randomly for each slide and the mean value was calculated.

B. Representative images of Periodic acid–Schiff (PAS) staining (scale bars: 100 μm) and statistical graph of the number of PAS^+^ goblet cells in the villus and crypt of WT mice and DR5^-/-^ mice (n=6). At least15 correctly aligned villi including crypts were counted randomly for each slide and the mean value was calculated.

C. Immunohistochemical staining of MUC2 to label the goblet cell (scale bars: 100 μm) and statistical graph of the number of MUC2^+^ goblet cells in villus and crypt of WT mice and DR5^-/-^ mice (n=6). At least 15 correctly aligned villi including crypts were counted randomly for each slide and the mean value was calculated.

**Figure S3 Representative images of TUNEL staining in ileum of WT mice and DR5^-/-^ mice (scale bars: 100 μm).**

The red arrows indicate the TUNEL^+^ differentiated epithelial cells in the villi.

**Figure S4 Bioymifi administration has no effect on the growth of organoid derived from DR5^-/-^ crypts**

Data were expressed as mean ± SEM. Statistical analyses were performed by the two-way ANOVA followed by Sidak's multiple comparisons test.

A. Representative images of intestinal organoids generated from ileal crypts of DR5^-/-^ mice treated with or without Bioymifi (100 nM) (scale bars: 100 μm). Photographs were taken 24 h, 48 h and 72 h after Bioymifi administration.

B. Quantitative analysis of the area, number of buds in Bioymifi (100 nM) treated and untreated organoids derived from crypts of DR5^-/-^ mice (n=6). Organoid area and budding were quantified according to the method shown in figure 4D.

**Figure S5 TRAIL silencing in organoids inhibits the gene expression of Lgr5, CDX2, Muc2 and Lyz**

qRT-PCR was performed to test the gene expression. Data were expressed as mean ± SEM. Unpaired t-test was performed to compare the difference between NC group and siTRAIL group (**P* < 0.05, ****P* < 0.001, n=5).

**Figure S6 Exogenous supplementation of Wnt3a improves the formation of organoid derived from DR5^-/-^ crypts**

Data were expressed as mean ± SEM. Statistical analyses were performed by the two-way ANOVA followed by Sidak's multiple comparisons test (**P* < 0.05, ***P* < 0.01, ****P* < 0.001, *****P* < 0.0001).

A. Representative images showing the effect of Wnt3a supplement (100 ng/ml) on organoids derived from crypts of DR5^-/-^ mice (scale bars: 100 μm).

B. The statistical graph showed the effect of Wnt3a supplementation on the area and budding of organoid derived from crypts of DR5^-/-^ mice (n=6). Organoid area and budding were quantified according to the method shown in figure 4D.

**Figure S7 PD0325901 has no effect on the formation of organoid derived from DR5^-/-^ crypts**

Data were expressed as mean ± SEM. Statistical analyses were performed by the two-way ANOVA followed by Sidak's multiple comparisons test.

A. Representative images to show the effect of PD0325901 (1 μM) on organoids derived from crypts of DR5^-/-^ mice (scale bars: 100 μm).

B. The statistical graph showing the effect of PD0325901 on the area and budding of organoid derived from DR5^-/-^ crypts (n=6). Organoid area and budding were quantified according to the method shown in figure 4D.
