# Supplementary figures and images for: Death receptor 5 is required for intestinal stem cell activity during intestinal epithelial renewal at homoeostasis
Source: Cell Death Dis. 2024 Jan 10;15(1):27. doi: 10.1038/s41419-023-06409-4 (PMC10782029; doi:10.1038/s41419-023-06409-4)

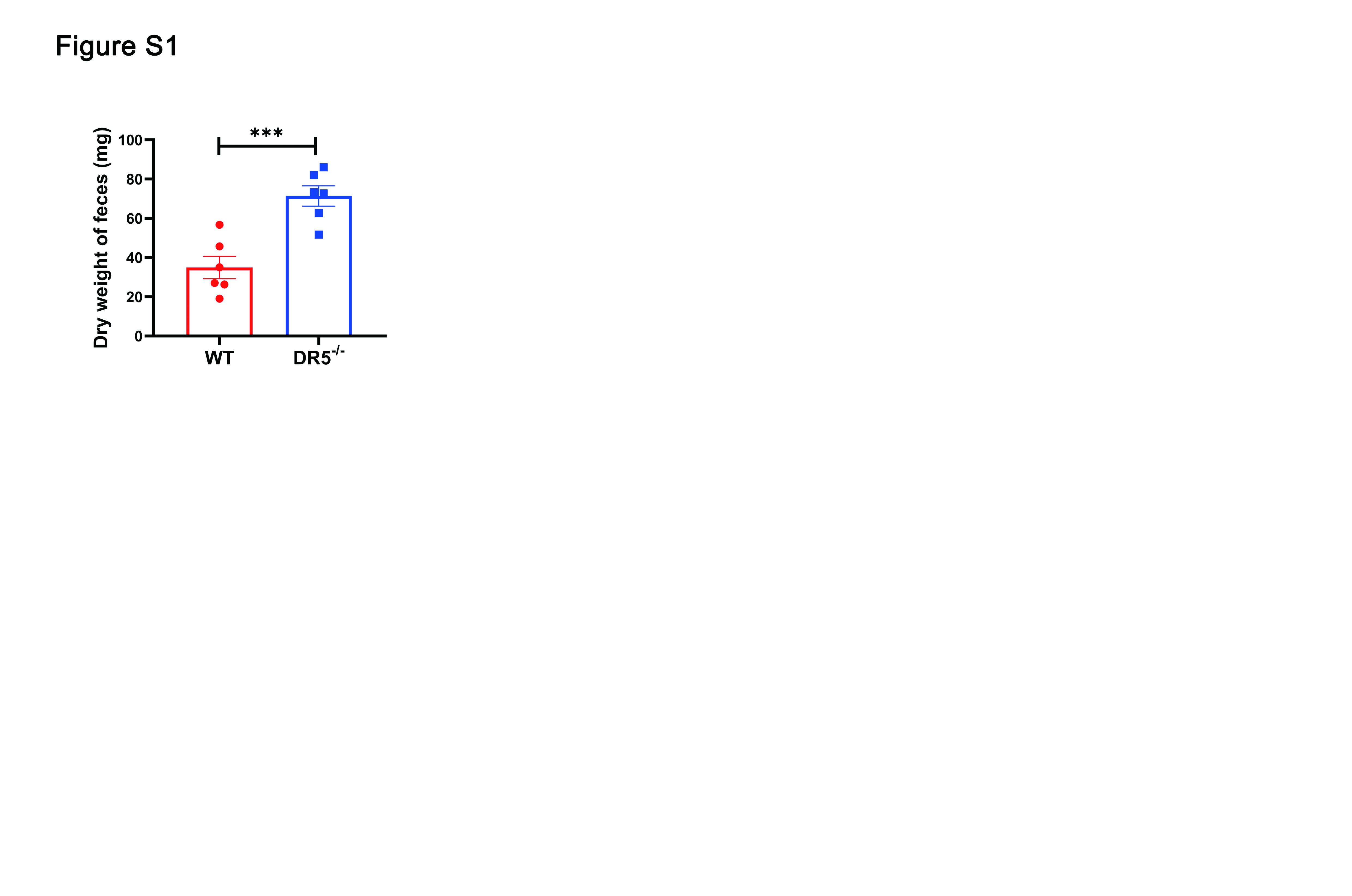

Supplement: Supplementary file 2 — Figure S1 [file 41419_2023_6409_MOESM2_ESM.tif]

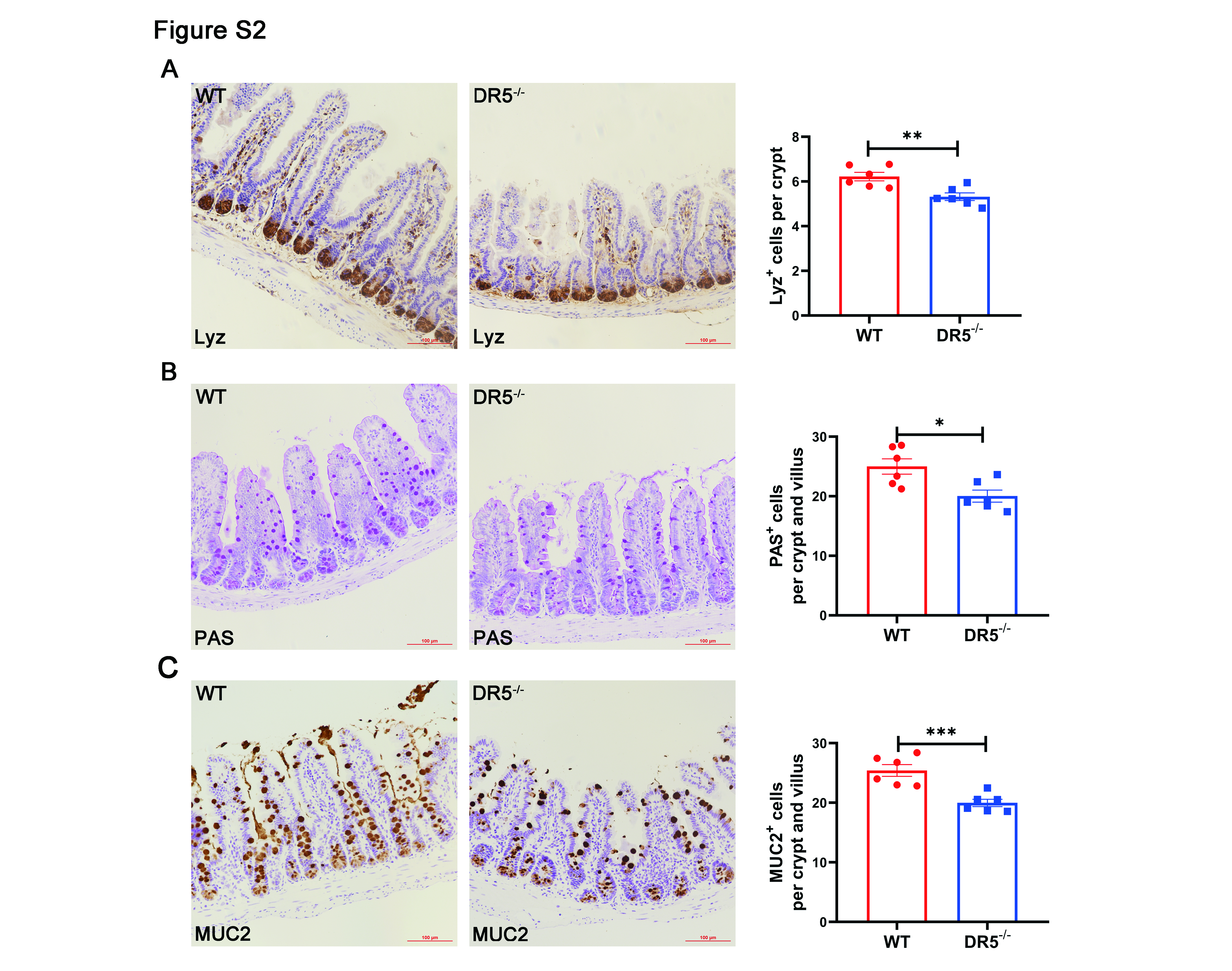

Supplement: Supplementary file 3 — Fgiure S2 [file 41419_2023_6409_MOESM3_ESM.tif]

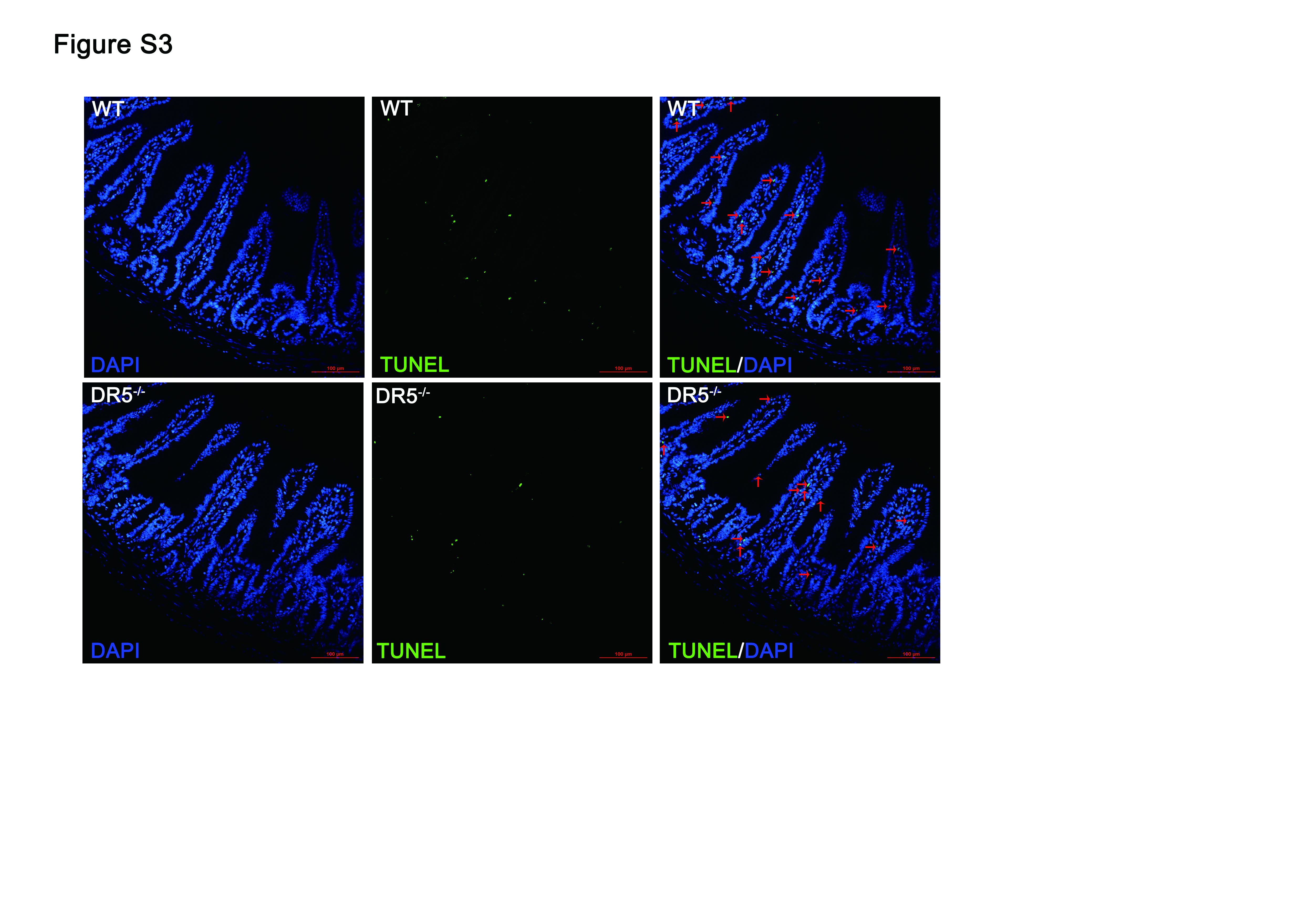

Supplement: Supplementary file 4 — Fgiure S3 [file 41419_2023_6409_MOESM4_ESM.tif]

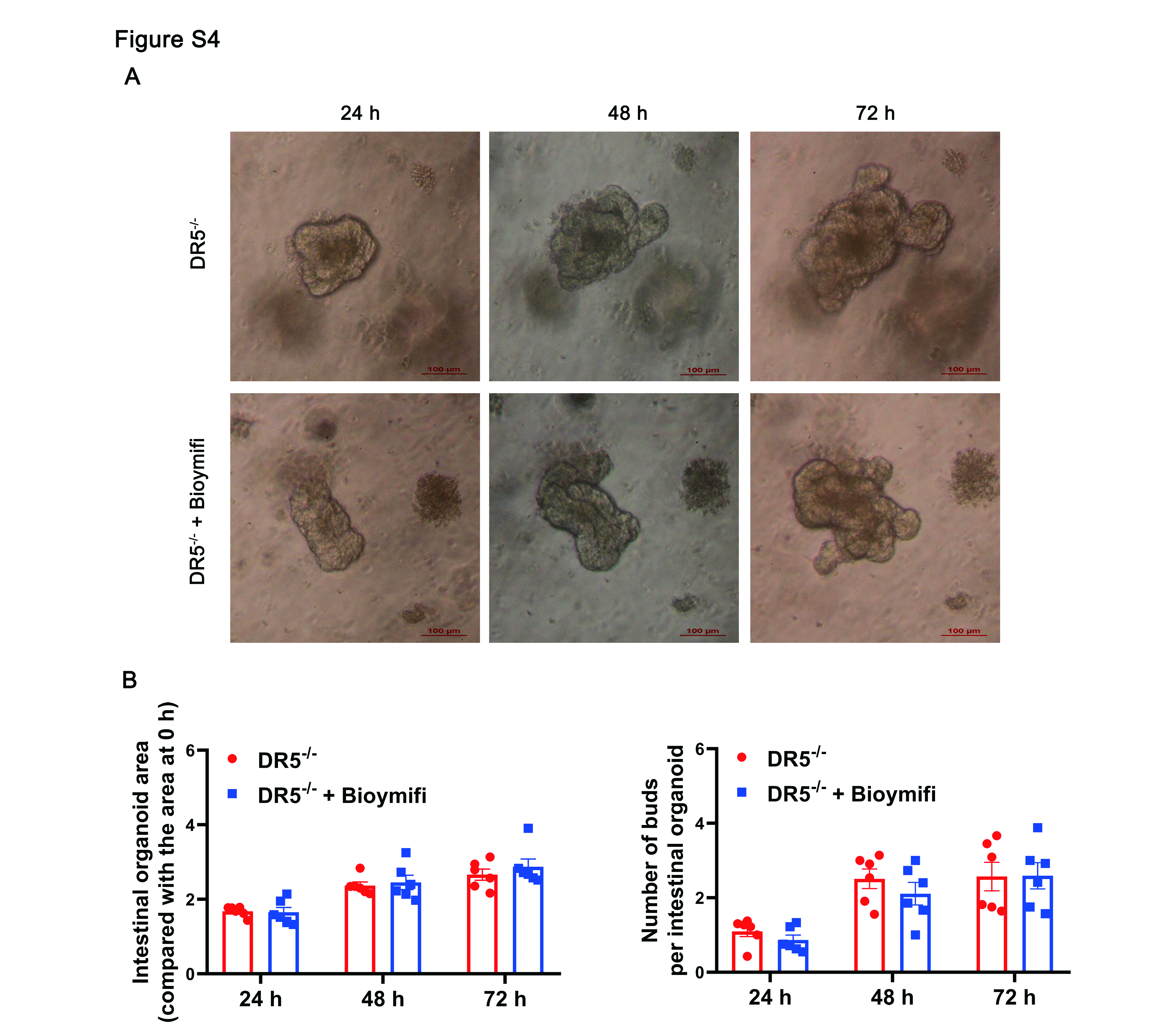

Supplement: Supplementary file 5 — Fgiure S4 [file 41419_2023_6409_MOESM5_ESM.tif]

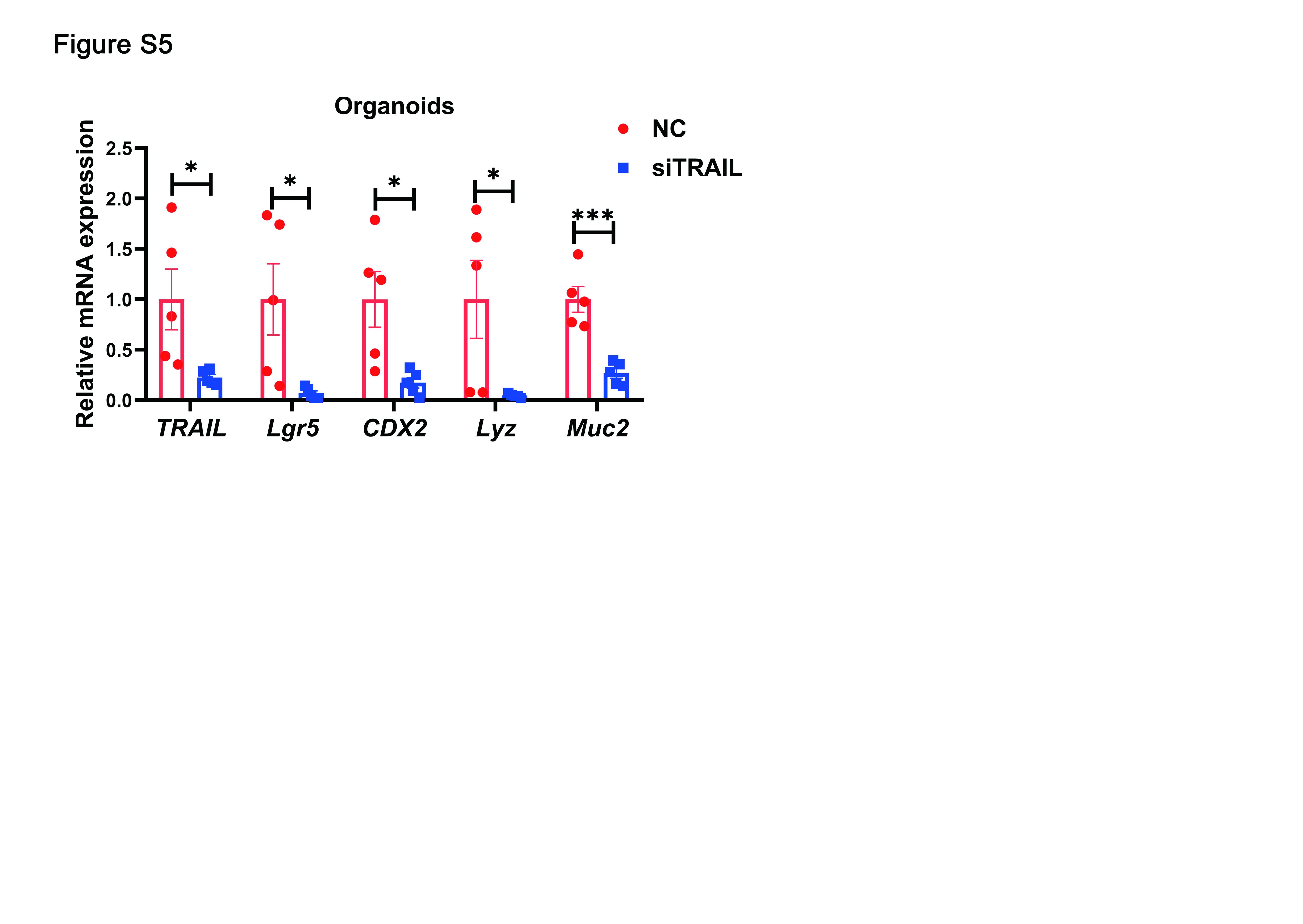

Supplement: Supplementary file 6 — Figure S5 [file 41419_2023_6409_MOESM6_ESM.tif]

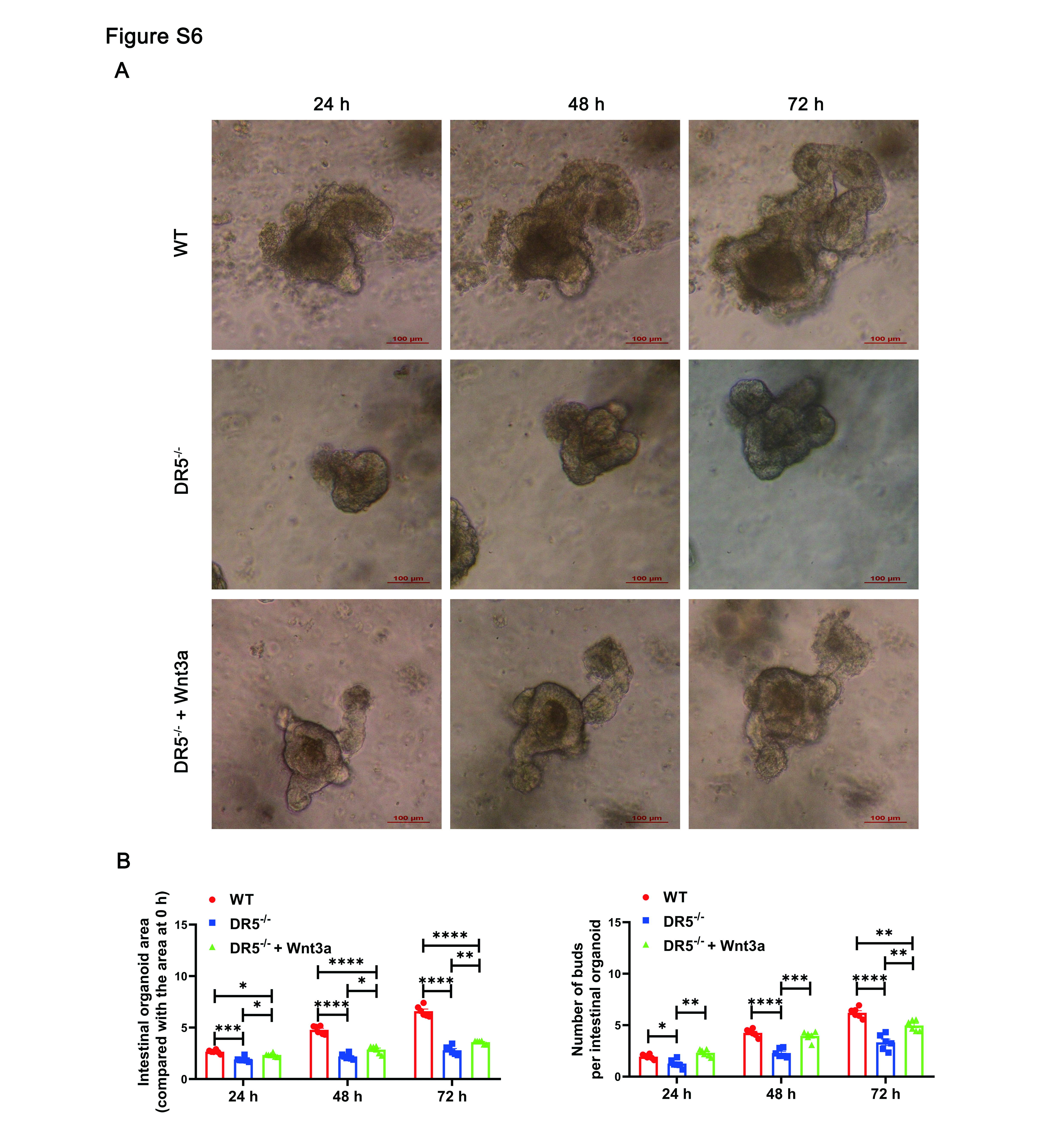

Supplement: Supplementary file 7 — Figure S6 [file 41419_2023_6409_MOESM7_ESM.tif]

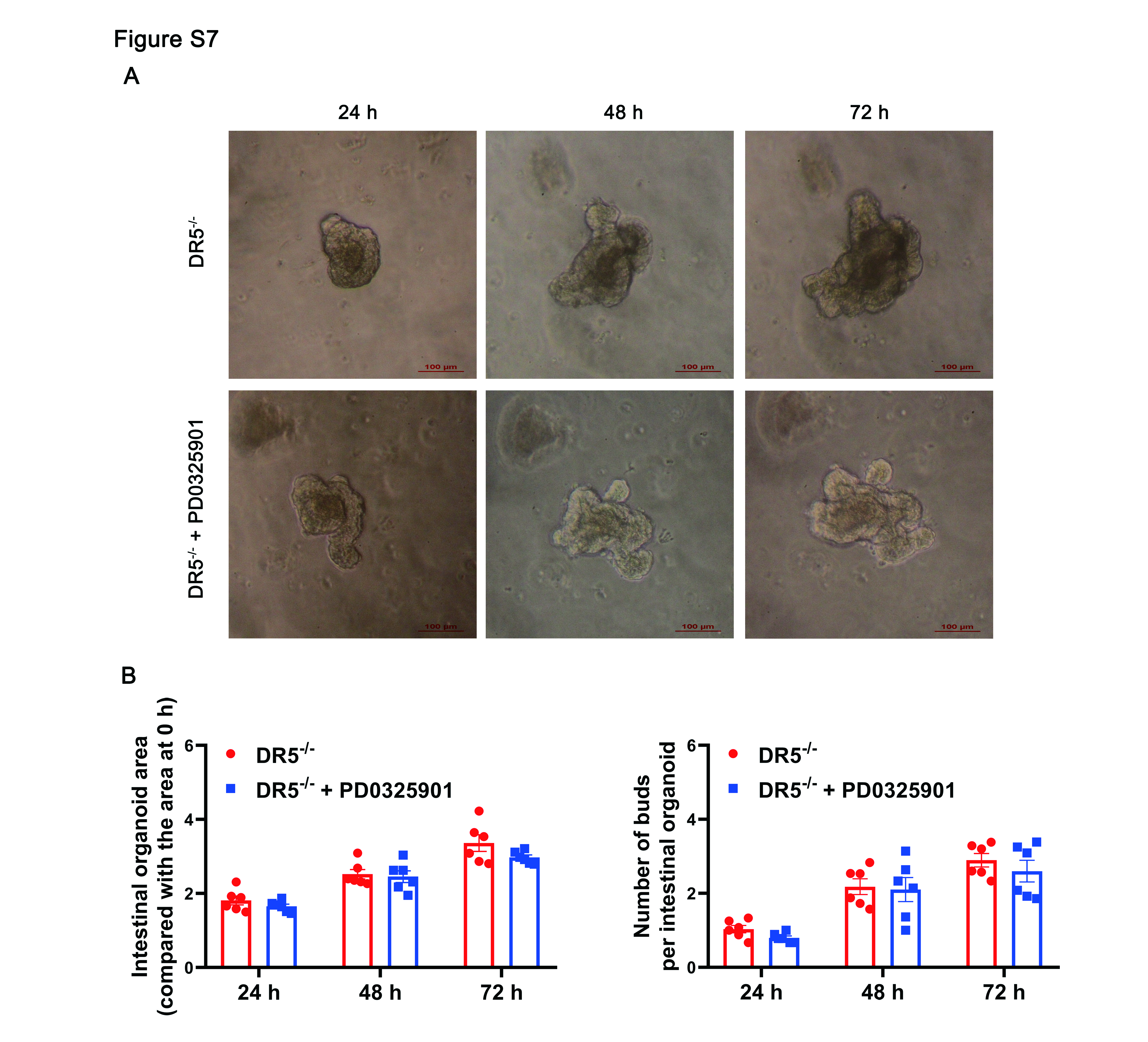

Supplement: Supplementary file 8 — Figure S7 [file 41419_2023_6409_MOESM8_ESM.tif]

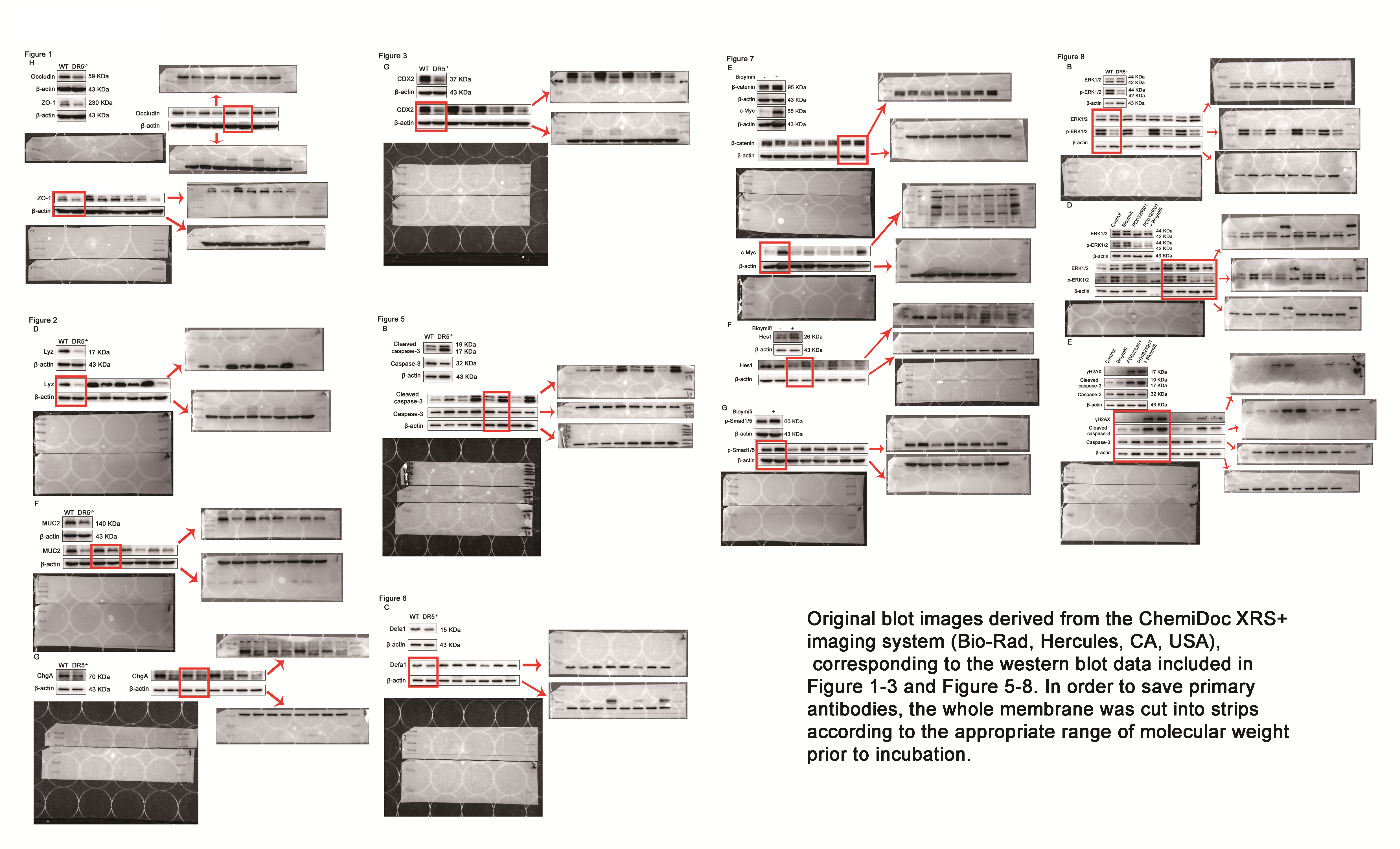

Supplement: Supplementary file 9 — Original data [file 41419_2023_6409_MOESM9_ESM.tif]
